# Impact of treatment and clinical characteristics on the survival of children with medulloblastoma in Mexico

**DOI:** 10.3389/fonc.2024.1376574

**Published:** 2024-05-02

**Authors:** Violeta Salceda-Rivera, Isidoro Tejocote-Romero, Diana S. Osorio, Rosalba Bellido-Magaña, Araceli López-Facundo, Susana E. Anaya-Aguirre, Daniel Ortiz-Morales, Roberto Rivera-Luna, Evelyne Reyes-Gutiérrez, Rebeca Rivera-Gómez, Liliana Velasco-Hidalgo, Deyanira Cortés-Alva, Sandra Lagarda-Arrechea, Farina E. Arreguín-González, Alma E. Benito-Reséndiz, Silvia Chávez-Gallegos, Eloy Pérez-Rivera, Guillermo J. Gaytán-Fernández, José A. León-Espitia, Jociela Domínguez-Sánchez, Carlos Leal-Cavazos, Citlalli Simón-González, Tania C. Larios-Farak, Nubia A. Macías-García, Ana C. García-Espinosa, Francisco Guerrero-Maymes, Paola Casillas-Toral, Oscar González-Ramella

**Affiliations:** ^1^ Hospital Civil de Guadalajara “Dr. Juan I. Menchaca”, Department of Pediatrics, Divisions of Pediatric Hematology-Oncology, Guadalajara, Jalisco, Mexico; ^2^ IMIEM, Instituto Materno Infantil del Estado de Mexico, Secretaria de Salud, Toluca, Estado de Mexico, Mexico; ^3^ Department of Pediatric Oncology, ISSEMYM, Instituto de Seguridad Social del Estado de México y Municipios, Toluca, Estado de Mexico, Mexico; ^4^ ICON PLC, Clinical Research Organization, Dublin, Ireland; ^5^ Hospital General Regional de Leon, Leon, Guanajuato, Mexico; ^6^ Instituto Mexicano del Seguro Social, Centro Médico Nacional La Raza, Mexico City, Mexico; ^7^ Department of Pediatric Oncology, Hospital General de México “Dr. Eduardo Liceaga”, Mexico City, Mexico; ^8^ Department of Pediatric Oncology, Hospital Militar de Especialidades de la Mujer y Neonatología, Mexico City, Mexico; ^9^ Department of Pediatric Oncology, Instituto Nacional de Pediatria, Mexico City, Mexico; ^10^ Hospital de Especialidades del Niño y de la Mujer, Secretaria de Salud, Queretaro, Mexico; ^11^ Hospital General de Tijuana, Universidad Autonoma de Baja California, Tijuana, Baja California, Mexico; ^12^ Hospital del Niño DIF Hidalgo, Sistema Nacional para el Desarrollo Integral de la Familia, Hidalgo, Mexico; ^13^ Department of Pediatric Oncology, Instituto Estatal de Cancerologia, Colima, Mexico; ^14^ Centro Médico Nacional “20 de Noviembre” del Instituto de Seguridad y Servicios Sociales de los Trabajadores del Estado (ISSSTE), Mexico City, Mexico; ^15^ Department of Pediatric Oncology, Hospital Infantil “Eva Samano de López Mateos”, Morelia, Michoacan, Mexico; ^16^ Department of Pediatric Oncology, Hospital Regional de Alta Especialidad del Bajío, Leon, Guanajuato, Mexico; ^17^ Department of Pediatric Oncology, Centenario Hospital Miguel Hidalgo, Aguascalientes, Mexico; ^18^ Hospital Universitario “Dr. José Eleuterio González”, Universidad Autónoma de Nuevo Leon, Nuevo Leon, Mexico; ^19^ Department of Pediatric Oncology, Hospital Regional de Alta Especialidad del Niño “ Dr. Rodolfo Nieto Padrón”, Tabasco, Mexico; ^20^ Department of Pediatric Oncology, Hospital Infantil del Estado de Sonora, Hermosillo, Sonora, Mexico; ^21^ Department of Pediatric Oncology, Hospital del Niño “Dr. Federico Gómez Santos”, Saltillo, Coahuila, Mexico; ^22^ Department of Pediatric Oncology, Hospital Infantil de Especialidades de Chihuahua, Chihuahua, Chihuahua, Mexico; ^23^ Department of Pediatric Oncology, Hospital General de Occidente, Zapopan, Jalisco, Mexico

**Keywords:** medulloblastoma, survival, clinical characteristics, low and middle income countries, CNS tumors, childhood, brain tumor

## Abstract

**Introduction:**

Data on medulloblastoma outcomes and experiences in low- and middle-income countries, especially in Latin America, is limited. This study examines challenges in Mexico’s healthcare system, focusing on assessing outcomes for children with medulloblastoma in a tertiary care setting.

**Methods:**

A retrospective analysis was conducted, involving 284 patients treated at 21 pediatric oncology centers in Mexico.

**Results:**

High-risk patients exhibited markedly lower event-free survival than standard-risk patients (43.5% vs. 78.3%, p<0.001). Influential factors on survival included anaplastic subtype (HR 2.4, p=0.003), metastatic disease (HR 1.9, p=0.001); residual tumor >1.5cm², and lower radiotherapy doses significantly impacted event-free survival (EFS) and overall survival (OS). Platinum-based chemotherapy showed better results compared to the ICE protocol in terms of OS and EFS, which was associated with higher toxicity. Patients under 3 years old displayed notably lower OS and EFS compared to older children (36.1% vs. 55.9%, p=0.01).

## 1 Introduction

Brain tumors are the most frequent solid tumors in children and adolescents, and they represent the major cause of cancer-related mortality in childhood. Medulloblastoma is the most common malignant brain tumor of childhood (1). However, there is very little data available in low- and middle-income countries (LMIC) regarding the outcome and, more importantly, the experience (2). Cancer registries in Latin America cover only 21% of the cases, compared to 99% in the USA and 86% in Canada (3). This demonstrates a problem in MIC, where implementing a national cancer registry system is challenging.

In Mexico, our closest data comes from single institutions or collaborations among a few hospitals, and in the best-case scenario, from one health system. In 2015, the incidence of intracranial neoplasms among children under 18 years treated with Popular Medical Insurance, which covers 55% of childhood cancer, was 10.3 cases per million/year (4, 5).

The healthcare system in Mexico, like other middle-income countries, faces several difficulties in delivering quality care (6, 7). In general, oncologists and patients struggle with the lack of accessible imaging resources such as MRI or CT scans, difficulties in initiating timely radiotherapy, limited availability of equipment like linear accelerators and 3D programming, saturated neurosurgery services, and a shortage of neurosurgeons trained in pediatrics (2, 8). Additionally, there are other important co-morbidity problems such as malnutrition, a high rate of infections that delay treatments, and the remote distances that some patients must travel to access oncology centers (9).

The improvement in the cure and quality of survival of children with medulloblastoma relies not only on chemotherapy protocols but also on multidisciplinary management, diagnostic technologies (such as MR imaging), radiation therapy, skilled neurosurgeons, radiotherapists, and board-certified pediatric oncologists.

The purpose of this study is to assess the outcomes of patients with medulloblastoma and their characteristics, as treated in a tertiary care setting in a middle-income country.

## 2 Methods

We conducted a retrospective analysis of the data from 284 patients who were treated between 1997 and 2017 at 21 pediatric oncology centers in Mexico.

For risk stratification, patients were divided into two prognosis groups, we used Chang Staging System to classify them as standard- and high-risk, as shown in **Table 1** (6). Treatment modalities included surgery, radiotherapy, the timing of treatments, the modality (cobalt vs. linear accelerator) used for radiotherapy, and the type of chemotherapy.

**Table 1:** Risk stratification of Medulloblastoma.

Standard risk (All of the following)	High risk (Any one of the following)
≥3 years of age	<3 years of age
Residual tumor <1.5cm^2^	Residual tumor >1.5cm^2^
Non-metastatic disease	Metastatic disease
Classic or desmoplastic histology	Large cell-anaplastic histology
Complete staging	Incomplete staging

### 2.1 Statical analyses

Descriptive data was presented in terms of frequencies and percentages, while quantitative data was described using mean, standard deviation, minimum, and maximum values. P-values less than 0.05 were considered significant. The prognostic value was assessed through multivariate analysis using the Cox regression model and the log-rank test. Nonparametric overall survival (OS) and event-free survival (EFS) were computed using Kaplan-Meier curves, and the log-rank test was employed to compare survival differences according to different variables. EFS was defined as the interval between the time of diagnosis and relapse or death. Data management and analysis were performed using SPSS version 23.0.

## 3 Results

A total of 284 patients from 21 pediatric oncology centers in Mexico, ranging in age from 1 month to 17 years old, were included in the study, with an average age at diagnosis of 6.7 ± 4.05 years old. Among the patients, 17.6% (n=50) were less than 3 years old. The male-to-female ratio was 1.6:1, and there was no significant difference in age or prognosis based on gender.

**Figure 1:**
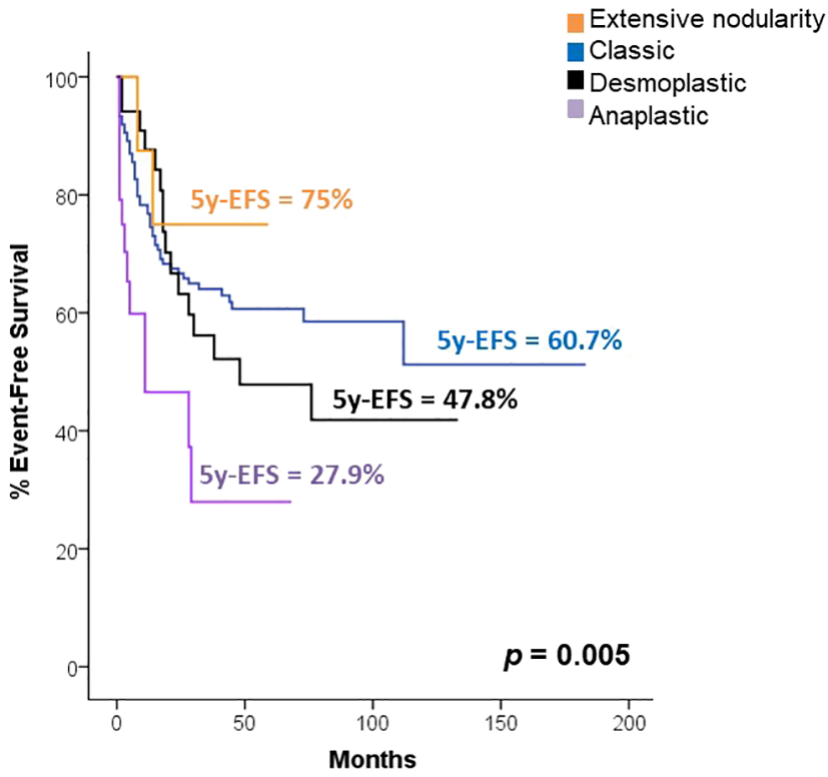
Survival according to risk classification.

Among all the patients, the most common pathology subtype was classic medulloblastoma (53.9%, n=153), followed by desmoplastic (12.7%, n=36), large cell-anaplastic (8.5%, n=24), and extensive nodularity (3.2%, n=9). However, in 21.8% (n=62), the pathology report did not specify the subtype. All patients with anaplasia were in the high-risk group, and in the rest of the different histologic groups no significant differences were found between high- and standard-risk patients (p >0.05). Survival analysis indicated that pathology subtype played a role in predicting survival, as children with anaplastic subtype had a 2.4 times higher risk of death or relapse compared to other subtypes (p=0.003). The 5-year EFS rates were 60.7% for classic type, 75% for extensive nodularity, 47.8% for desmoplastic, and 27.9% for anaplastic (p=0.005), as depicted in **Figure 1**.

**Figure 2:**
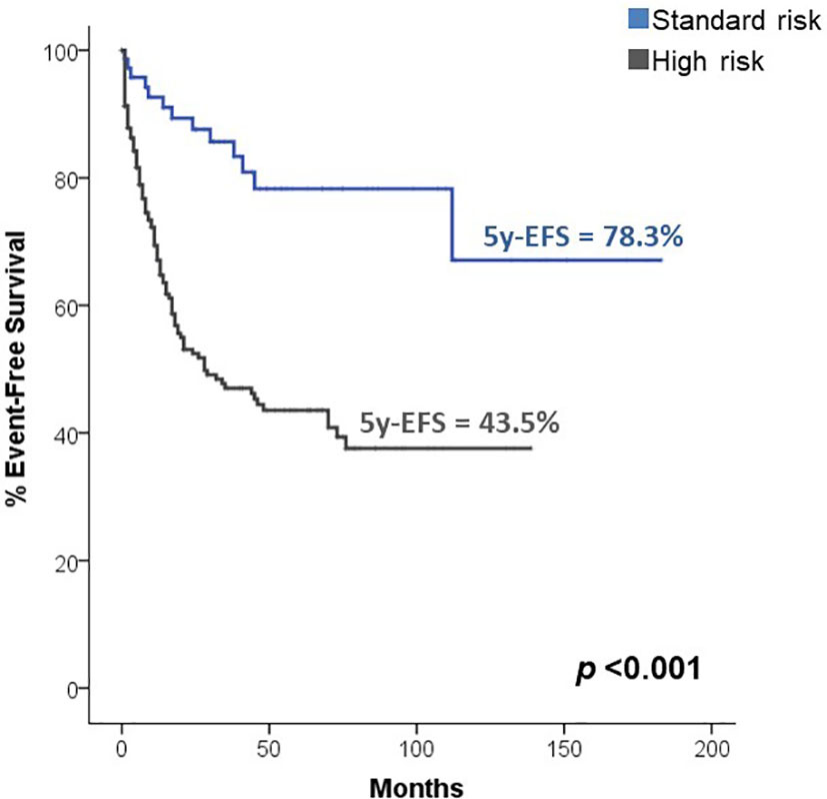
Survival according to histology.

Regarding the risk stratification of medulloblastoma, we found that 74.3% (n=211) of the patients were classified as high risk, while only 25.7% (n=73) were categorized as standard risk. Patients with high-risk demonstrated significantly lower EFS compared to patients with standard risk (5-year EFS 43.5% vs. 78.3%, p<0.001), as shown in **Figure 2**. Furthermore, having high-risk characteristics increased the risk of death or relapse by 3.7 times (p<0.001, 95% CI 2.11-6.72). **Table 2** describes the chemotherapy protocols that were used in high- and standard-risk patients, and **Table 3** describes the doses of radiotherapy used in both groups of patients.

**Table 2:** Chemotherapy protocol according to risk group.

	High risk n= 211	Standard risk n= 73	*p* value
Chemotherapy			
ICE protocol Carboplatin + VP-16 + VCR ± CPM Cisplatin + VCR ± VP-16 ± CPM Other regimens Without chemotherapy Unknown	130 (61.6%) 11 (5.2%) 33 (15.6%) 9 (4.3%) 17 (8.1%) 11 (5.2%)	24 (32.8%) 17 (23.2%) 26 (35.6%) 2 (2.7%) 3 (4.1%) 1 (1.3%)	<0.001 <0.001 0.001 0.461 0.303 0.308

**Table 3:** Radiotherapy doses based on clinical characteristics.

Characteristic	Posterior fossa Mean ± SD	Craniospinal Mean ± SD
All patients	52.1 ± 6.2 Gy	29.1 ± 7.8 Gy
High risk	51.7 ± 7.1 Gy	30.4 ± 7.6 Gy
Standard risk	53.01 ± 3.6 Gy	26.5 ± 7.4 Gy
>3 years old	53.1 ± 3.9 Gy	29.05 ± 7.7 Gy
<3 years old	43.8 ± 13.4 Gy	29.8 ± 8.3 Gy

Based on the approach for metastasis, utilizing cerebrospinal fluid cytology and MRI, 54.9% (n=156) of the patients did not show metastasis at diagnosis (M0). Microscopic evidence of tumor cells in cerebrospinal fluid (M1) was observed in 15.8% of cases, while 9.9% showed intracranial metastasis (M2), 11.3% had gross nodular seeding of spinal metastasis (M3), and 1.8% had metastasis outside the central nervous system (M4). Patients with metastatic disease had a 1.9 times higher risk of death (p=0.001, 95% CI 1.29-2.89).

Since residual tumor after surgical resection is considered part of the risk stratification, we performed an analysis of the surgical results. Based on post-operative CT or MRI, residual tumor less than 1.5cm^2^ was achieved in 46.1% (n=131) of the patients, and gross tumor resection was accomplished in 29.6% (n=84) of the cases. Survival analysis revealed that patients with a residual tumor less than 1.5cm^2^ and gross tumor resection had significantly higher EFS compared to those with residual tumor >1.5cm^2^ (5-year EFS 72.1% vs. 33.6%, p<0.001). Further statistical analysis showed that a residual tumor >1.5cm^2^ increased the risk of death or relapse by 3.6 times (p<0.001). Within the first 48 hours post-surgery, a CT or MRI was obtained in 62.7% (n=178) of the cases. Interestingly, only 8.5% (n=24) of the children displayed clinical data of cerebellar mutism syndrome.

In the entire cohort craniospinal radiotherapy (CSI) was administered to 75% (n=213) of the patients; conformal, intensity-modulated and, in some centers, cobalt-based radiotherapy was used; all patients received posterior fossa boost. **Table 3** provides an overview of radiotherapy doses based on different clinical characteristics. We found that the dose to posterior fossa radiation impacted OS, with a 3-year OS of 81.8% for patients who received >50Gy and 60.2% for those who received <50Gy (p=0.04). The mean age at which patients received radiotherapy was 7.4 ± 3.6 years, ranging from 1 to 17 years old. Notably, 9.7% (n=20) of the patients who received CSI were less than 3 years old. Of the cohort, 60 patients did not receive radiotherapy for various reasons such as lack of resources, age of the patient, or early death due to complications. Of this group of patients, 26 were younger than three years, and the mean age was 5.2 years. The patients who did not receive radiotherapy had a 1-year OS of 36.7% and a 3-year OS of 19.3%.

In 31.3% (n=89) of the cases, radiotherapy was applied after surgical resection, and in 21.8% (n=62) of the children was initiated within the first 6 weeks after surgery. No significant differences in the risk of death or relapse were found between those who initiated radiotherapy within 6 weeks and those who had a delay of more than 6 weeks (p=0.717). In the case of patients who took more than 6 weeks to receive radiotherapy after surgery, this was due firstly to infectious or post-surgical complications, and secondly to administrative problems such as availability, equipment failure or economic issues.

Because of the wide variability among healthcare systems in Mexico, this study found that different chemotherapy regimens were used. The most frequently used regimen was the ICE regimen (ifosfamide, carboplatin, and etoposide), with a median of 7 cycles, followed by protocols based on cisplatin such as the Packer protocol (10), or based on carboplatin protocols (11). In a very low frequency, other regimens such as irinotecan, temozolomide, or nitrosoureas-based protocols were utilized.

**Figure 3:**
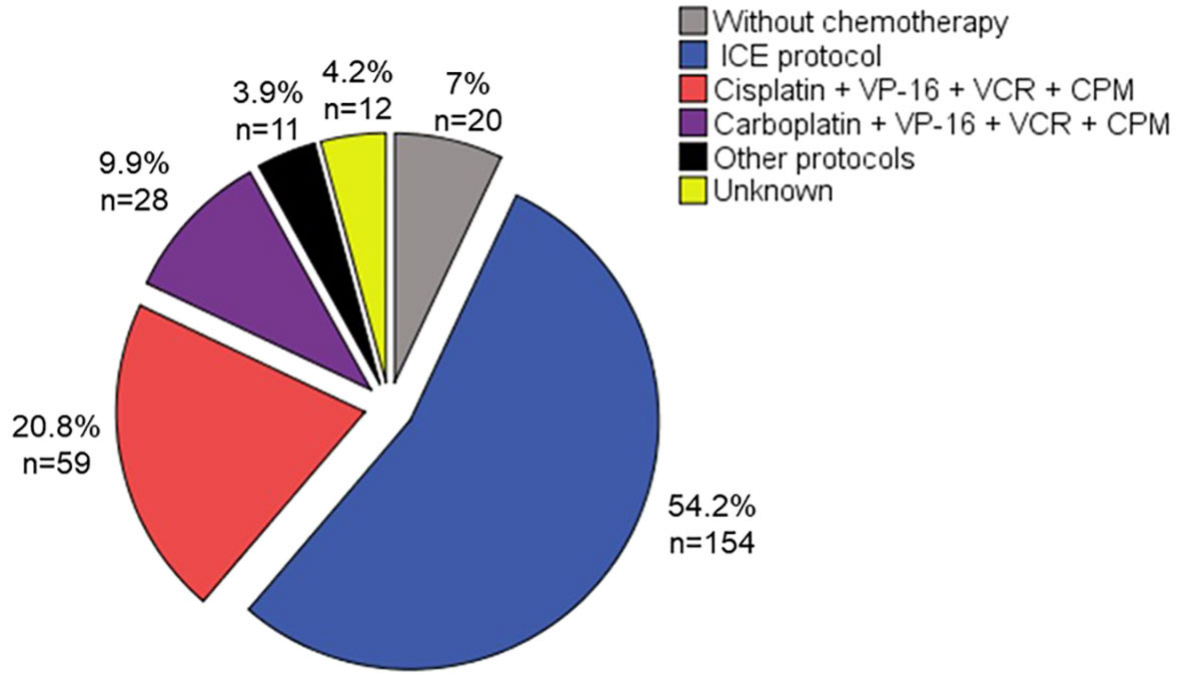
Chemotherapy regimens used.

Some patients were reported as not receiving chemotherapy. This was either due to their arrival in precarious health conditions that led to death before any treatment could be administered or due to expiration resulting from post-surgical complications. **Figure 3** provides an overview of the frequency and percentage of the different chemotherapy regimens used.

**Figure 4:**
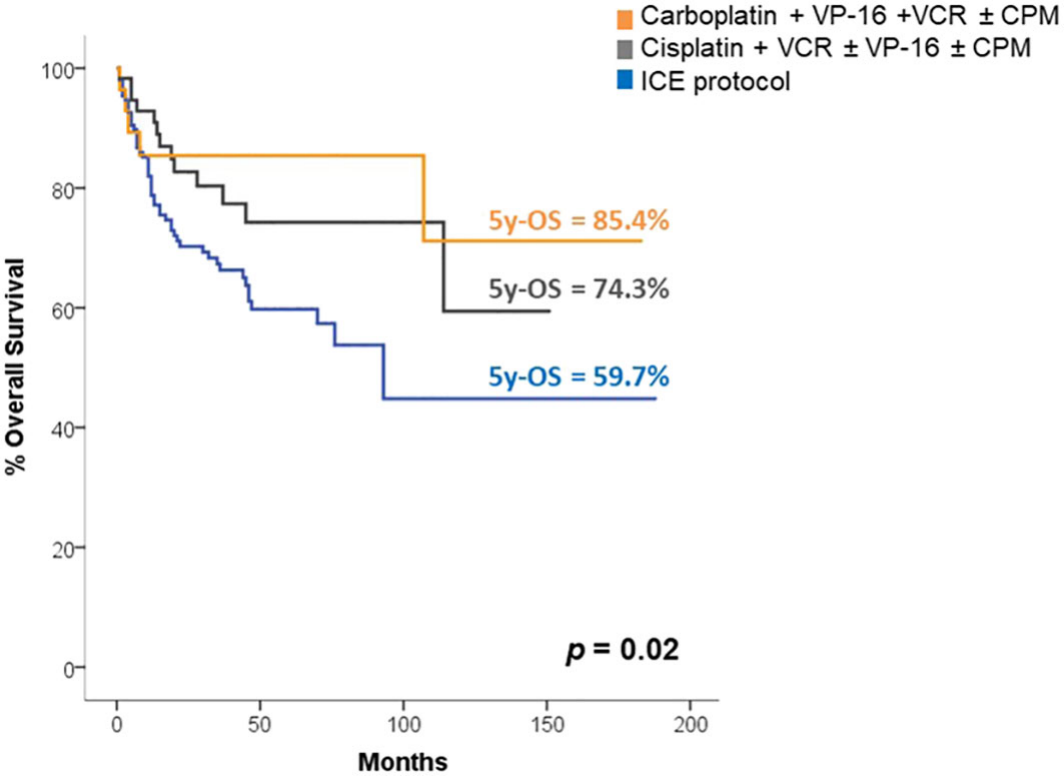
Survival according to chemotherapy regimens.

The platinum-based regimens demonstrated superior OS and EFS compared to the ICE protocol, with 5-year OS rates of 73.6% vs. 59.7% (p = 0.029) and 5-year EFS rates of 63% vs. 53.6% (p=0.040), respectively, as is shown in **Figure 4**. Through multivariate analysis to predict the risk of death or relapse, we found that the use of the ICE protocol was associated with a 1.7 times higher risk of death or relapse compared to the use of any other chemotherapy regimen (p=0.032), mainly explained by toxicity complications.

Regarding the survival analyses of the entire cohort, the 5-year OS and EFS rates were found to be 59.9% and 52.6% respectively. **Table 4** presents the results, highlighting significant differences in OS and EFS based on various patient characteristics, including age, histology, and risk. **Table 5** provides a description of the factors that influenced death or relapse.

**Table 4:** Survival according to different characteristics.

	3y-OS	5y-OS	*p* value	3y-EFS	5y-EFS	*p* value
**High risk**	56.9%	52.6%	<0.001	47%	43.5%	<0.001
**Standard risk**	85.7%	80.6%		85.7%	78.3%	
**<3 years old**	47.3%	47.3%	0.04	36.1%	36.1%	0.011
**>3 years old**	68.3%	62.5%		61.5%	55.9%	
**Anaplastic**	52.2%	52.2%	0.011	27.9%	27.9%	0.001
**Other histology**	68.1%	63.9%		63%	58.7%	
**Residual tumor >1.5cm^2^**	46.6%	44.7%	<0.001	37.3%	33.6%	<0.001
**Residual tumor <1.5cm^2^**	82.5%	77.4%		76.9%	72.1%	
**Metastatic disease**	53.6%	50.9%	0.001	45%	42.4%	<0.001
**Non-metastatic disease**	73.6%	67.4%		67.2%	61%	

**Table 5:** Characteristics related to death or relapse by multivariate regression.

Factor related to death or relapse	Hazard ratio (risk)	*p* value	95% CI
ICE protocol	1.7	0.032	1.04-2.81
Metastatic disease	1.9	<0.001	1.33-2.8
Anaplastic	2.4	0.003	1.35-4.36
Residual tumor >1.5cm^2^	3.6	<0.001	2.35-5.53
High risk	3.7	<0.001	2.11-6.72

The group of patients under 3 years old, exhibited significantly lower OS and EFS compared to older patients (5-year EFS 36.1% vs. 55.9%, p=0.01). The type of chemotherapy they received is described in **Table 6**, with the ICE protocol being the most used. Only two patients received autologous stem cell transplant, both with minimal residual disease. One of them is alive with 17 months of follow-up and received focal radiotherapy, while the other one did not receive radiotherapy and passed away after 21 months of diagnosis.

**Table 6:** Chemotherapy used in children under 3 years.

	High risk n= 211	Standard risk n= 73	*p* value
Chemotherapy			
ICE protocol Carboplatin + VP-16 + VCR ± CPM Cisplatin + VCR ± VP-16 ± CPM Other regimens Without chemotherapy Unknown	25 (50%) 4 (8%) 10 (20%) 2 (4%) 4 (8%) 5 (10%)	24 (32.8%) 17 (23.2%) 26 (35.6%) 2 (2.7%) 3 (4.1%) 1 (1.3%)	<0.001 <0.001 0.001 0.461 0.303 0.308

## 4 Discussion

Medulloblastoma is a tumor that predominantly occurs in pediatric age, with most cases diagnosed between 5 and 10 years (12). Our study found a similar median age of 6.0 years (SEM 0.24), aligning with previous findings.

Similar to a study conducted by Akyüz et al. in Turkey (13), we observed a male-to-female ratio of 1.6. The relationship between gender and survival has been a subject of discussion. Unlike the results reported by Curran et al. from the U.S. Surveillance Epidemiology and End Results (SEER-9) registry (14), we did not find a difference in OS or EFS based on gender.

In our study, we observed the following frequencies of histological variants: classic medulloblastoma 53.9%, desmoplastic 12.7%, large cell-anaplastic 8.5%, and extensive nodularity 3.2%. These findings are very similar to the report by Louis DN et al., who found 72% classic, 14% desmoplastic, 11% large cell/anaplastic, and 3% extensive nodularity (12).

Regarding survival, one unexpected result was observed in desmoplastic/extensive nodularity histology, which is known for its nodular architecture and excellent prognosis (15–17). Even without radiotherapy as adjuvant treatment, children younger than 3 years with desmoplastic histology showed a 10-year progression-free survival of 85%, compared to classic histology with 34% (18). However, in our study, the survival of the desmoplastic variant was similar to the classic variant (5-year OS 58.7% vs. 52.2%). The patients with extensive nodularity variant showed an excellent outcome with a 5-year OS of 83.3%, which is similar to the prognosis reported for this histological variant in other studies (19). Another unexpected result was the lower frequency of desmoplastic/extensive nodularity histology of 24% among our 50 patients under three years old, while other series reported a frequency of approximately 44% for the desmoplastic variant in patients under three years old (20). We believe that these results can also be explained by a recurring issue we encountered wherein: 21.8% of our patients, the histological variant was not reported in the pathology results. Additionally, since we do not routinely perform molecular studies, we are unaware of the frequency of mutations that confer a worse prognosis to the sonic hedgehog subgroup, such as TP53 mutations or specific chromosomal aberrations (19).

Regarding the large cell-anaplastic histological subtype and survival, we found that it is a risk factor for death or relapse, with a hazard risk of 2.4, which is consistent with the findings of Eberhart et al., who reported that severe anaplasia alone is associated with worse clinical outcomes (p=0.002) (21). Other reports suggest that the anaplastic subtype is related to an inferior prognosis when certain biological characteristics are present, such as c-myc amplification (22). Unfortunately, we do not have information on the molecular markers of our patients, this is due to the fact that these studies are not available in our country.

In our cohort, the median dose for the posterior fossa radiotherapy boost was 52.1 ± 6.2 Gy, which is not significantly different from the recommended dose of 54 Gy (23). Comparing survival between patients who received less than 50 Gy and those who received more than 50 Gy, we found a significantly lower survival in the group that received a lower dose (5-year OS 52.6% vs. 76.7%, p=0.04). Similar reports by Silverman CL et al. (24) have associated the dose of radiotherapy received with survival, and another study by Santos MA et al. found a correlation between lower doses and poorer survival (5-year OS 80% vs. 58%, p=0.02), although they used a censored dose of 44 Gy (25). This underscores the importance of radiotherapy as a fundamental part of medulloblastoma treatment, as the tumor is known to be radiosensitive.

Among our 50 patients under 3 years old, 20 of them underwent irradiation. Of those, five patients underwent surgery, followed by radiotherapy and then chemotherapy, resulting in a 5-year OS of 80%. Fifteen patients after surgery received chemotherapy and then radiotherapy, with a 5-year OS of 83.1%. In contrast, those who did not receive radiotherapy had a significantly lower 5-year OS of 25.7% (p < 0.001). These results differ from a study by Rivera-Luna R. et al. (26), conducted with Mexican patients from different hospitals. In their series of 49 patients under 3 years old, 100% of those who received only chemotherapy died, while those who received chemotherapy and radiotherapy had a 5-year progression-free survival of 66%. It is crucial to explore other treatment strategies for these patients, such as intraventricular therapy or high-dose chemotherapy with hematopoietic progenitor cell rescue (18, 27, 28), to improve survival rates in Mexico. Although medulloblastoma is radiosensitive, CSI should be avoided in children under 3 years of age, due to its adverse effects that can be catastrophic at this age (29–31), other treatment strategies should be used for patients in this age group, especially when patients have desmoplastic nodular histology (17, 27, 32), since the objective of pediatric oncology is not only to cure but to achieve the best possible quality of life.

In our entire cohort, 17.6% (n = 50) of the patients were under 3 years old, and among them, 27 patients experienced death or relapse, resulting in a 5-year EFS of 36.1%. This finding is comparable to several reports that associate being under 3 years of age with a poor prognosis (26, 27). One of the reasons for this is the preference to avoid or delay radiotherapy in these patients due to the side effects associated with it (18).

In the analysis of survival according to chemotherapy regimen, we found that those based on carboplatin had the highest OS and EFS in our patients, with a 5-year OS of 85.4% and 5-year EFS of 68.1%. These results are very similar to the findings in pediatric patients in Cairo, where the regimen of carboplatin, etoposide, and vincristine led to a 5-year OS of 89% and disease-free survival at 5 years of 78% (33).

In our study, the regimens based on cisplatin showed the second-best survival, with a 5-year OS of 74.3%. This is consistent with results from a study in Turkey, a middle-income country, conducted by Akyüz et al., who reported an 8-year OS of 60% with the cisplatin plus etoposide regimen [16]. They transitioned to this regimen in an effort to improve survival and decrease the toxicity associated with their previous lomustine-based regimen, which showed an 8-year OS of 41.1%. However, our study’s survival with cisplatin-based regimens differs significantly from that reported in high-income countries. Such as Packer et al. reported a 5-year OS of 87% in standard-risk patients treated with the Children’s Oncology Group trial A9961, which included CSI therapy followed by adjuvant chemotherapy with cisplatin, vincristine and cyclophosphamide (10). Similarly, in high-risk patients treated with platinum-based chemotherapy, Tarbell et al. reported a 5-year OS of 76.1% (34).

Analyzing one of the reasons why the ICE protocol in this study is significantly associated with lower OS than other protocols, we found that it is associated with a high rate of toxicity, as described by Kanamori M et al. (35, 36). They reported adverse effects of ICE combination chemotherapy in the treatment of various brain tumors, including grade 4 neutropenia in 81.4% of cases, grade 4 thrombocytopenia in 35.4%, and infection in 26.8%, among other toxicities such as grade 4 anemia and elevated alanine and aspartate aminotransferases. These findings suggest that the high rate of adverse effects requires close follow-up or dose reduction.

One early complication observed within the first two days after surgical resection of medulloblastoma in children is cerebellar mutism syndrome. In our population, we found a low frequency of 8.5%, compared to the 24% reported by Robertson et al. (37). This difference in rates can be explained by our lack of intentional use of a diagnostic tool. In their study, Robertson et al. used a questionnaire aimed at identifying the presence and severity of cerebellar mutism syndrome.

In our total cohort, patients with standard-risk characteristics have been successfully treated, while the prognosis for children with high-risk characteristics remains poor. **Table 7** compares our survival rates with treatment protocols that use similar resources to those currently available in our country.

**Table 7:** Comparison of 5y-EFS according to risk.

Standard Risk		High Risk	
Mexico (this study)	78.3%	Mexico (this study)	43.5%
Gajjar et al., 2006 (SJMB96) (28)	83%	Gajjar et al., 2006 (SJMB96) (28)	70%
Packer et al., 1999 (CCG9892) (38)	79%	Zeltzer et al., 1999 (CCG921) (39)	63%
Packer et al., 2013 (COG A9961) (10)	81%	Jakacki et al., 2012 (40)	71%

## 5 Conclusion

This study has several strengths, including the collaboration of twenty-one pediatric oncology centers and a significant sample size from different regions of Mexico. However, it is important to note that this is a retrospective study, and future multi-institutional prospective clinical trials are needed to further define survival, risk factors, and outcomes in Mexico.

Managing medulloblastoma poses challenges in low and middle-income countries. Nevertheless, this study identifies characteristics that increase the risk of death in our patients. With feasible changes, we can improve staging and better guide treatment decisions, such as requesting histopathological subtyping and establishing direct communication with the radiotherapy team to discuss and determine the appropriate radiation dose for each patient, avoiding CSI in young children.

One of the most important aspects that we need to improve is our infrastructure in all cancer care centers for children with cancer, such as access to conformal radiotherapy, magnetic resonance imaging, neuronavigation, or microscopes for neurosurgery, to mention a few examples that would make significant improvements in survival. In addition, given the crucial role of genotype knowledge in medulloblastoma treatment worldwide, countries like Mexico should implement this important tool as part of routine practice to ensure accurate treatment for these children.

Implementing twinning programs, which have shown success in improving survival rates in low and middle-income countries, could also be a valuable strategy to consider (8, 41).

Additionally, it would be beneficial to develop unified national treatment guidelines and explore new treatment strategies for patients with high-risk disease and young children and consider using the least toxic chemotherapy protocols whenever possible, aiming to heal with the best possible quality of life.

## Data availability statement

The original contributions presented in the study are included in the article/supplementary material. Further inquiries can be directed to the corresponding author.

## Author contributions

VS-R: Conceptualization, Data Curation, Formal analysis, Investigation, Methodology, Project administration, Supervision, Validation, Visualization, Writing – original draft, Writing – review & editing. IT-R: Data Curation, Investigation, Validation, Visualization, Writing – original draft, Writing – review & editing. DO: Data Curation, Investigation, Validation, Visualization, Writing – original draft, Writing – review & editing. RB-M: Data Curation, Investigation, Validation, Visualization, Writing – original draft, Writing – review & editing. AL-F: Data Curation, Investigation, Validation, Visualization, Writing – original draft, Writing – review & editing. SA-A: Data Curation, Investigation, Validation, Writing – original draft, Writing – review & editing. DO-M: Data Curation, Investigation, Writing – original draft, Writing – review & editing. RR-L: Data Curation, Investigation, Validation, Writing – original draft, Writing – review & editing. ER-G: Data Curation, Investigation, Writing – original draft, Writing – review & editing. RR-G: Data Curation, Investigation, Writing – original draft, Writing – review & editing. LV-H: Data Curation, Investigation, Validation, Writing – original draft, Writing – review & editing. DC-A: Data Curation, Investigation, Writing – original draft, Writing – review & editing. SL-A: Data Curation, Investigation, Writing – original draft, Writing – review & editing. FA-G: Data Curation, Investigation, Writing – original draft, Writing – review & editing. AB-R: Data Curation, Investigation, Validation, Writing – original draft, Writing – review & editing. SC-G: Data Curation, Investigation, Writing – original draft, Writing – review & editing. EP-R: Data Curation, Investigation, Writing – original draft, Writing – review & editing. GG-F: Data Curation, Investigation, Writing – original draft, Writing – review & editing. JL-E: Data Curation, Investigation, Writing – original draft, Writing – review & editing. JD-S: Data Curation, Investigation, Writing – original draft, Writing – review & editing. CL-C: Data Curation, Investigation, Writing – original draft, Writing – review & editing. CS-G: Data Curation, Investigation, Writing – original draft, Writing – review & editing. TL-F: Data Curation, Investigation, Writing – original draft, Writing – review & editing. NM-G: Data Curation, Investigation, Writing – original draft, Writing – review & editing. AG-E: Data Curation, Investigation, Writing – original draft, Writing – review & editing. FG-M: Data Curation, Writing – original draft, Writing – review & editing. PC-T: Writing – original draft, Writing – review & editing. OG-R: Data Curation, Investigation, Writing – original draft, Writing – review & editing.
